# Can the electrophysiological action of rosiglitazone explain its cardiac side effects?

**DOI:** 10.1186/1471-2210-11-S2-A54

**Published:** 2011-09-05

**Authors:** Valéria Kecskeméti, Andrea Szebeni, Norbert Szentandrássy, Péter P Nánási

**Affiliations:** 1 Department of Pharmacology and Pharmacotherapy, Semmelweis University, 1089 Budapest, Hungary; 2 Department of Physiology, University of Debrecen, 4012 Debrecen, Hungary

## Background

Several recent clinical trials showed that the antidiabetic drug rosiglitazone is associated with an excess risk of cardiovascular adverse events. In spite of its widespread clinical application there is little information on its cellular cardiac effects in larger mammals. In the present study, therefore, concentration-dependent effects of rosiglitazone on action potential morphology and the underlying ion currents were studied in canine hearts.

## Methods

Standard microelectrode techniques, conventional whole cell patch-clamp, and action potential voltage-clamp techniques were applied in enzymatically dispersed ventricular cells.

## Results

At concentrations ≥ 10 µM rosiglitazone decreased the amplitude of phase-1 repolarization, reduced the maximum velocity of depolarization and caused depression of the plateau potential. These effects developed rapidly and were readily reversible upon washout. Rosiglitazone suppressed several transmembrane ion currents in a concentration-dependent manner under conventional voltage-clamp conditions and altered their kinetic properties. The EC_50_ value for this inhibition was 25.2 ± 2.7 µM for the transient outward K^+^ current (I_to_), 72.3 ± 9.3 µM for the rapid delayed rectifier K^+^ current (I_Kr_), and 82.5 ± 9.4 µM for the L-type Ca^2+^ current (I_Ca_) with Hill coefficients close to unity. The inward rectifier K^+^ current (I_K1_) was not affected by rosiglitazone up to concentrations of 100 µM. Suppression of I_to_, I_Kr_, and I_Ca_ was confirmed under action potential voltage-clamp conditions as well.

## Conclusions

The observed alterations in the densities and kinetic properties of ion currents may carry serious proarrhythmic risk in case of overdose intoxication with rosiglitazone, especially in older diabetic patients having multiple cardiovascular risk factors.

